# Proposal for a recovery prediction method for patients affected by acute mediastinitis

**DOI:** 10.1186/1749-7922-7-11

**Published:** 2012-05-10

**Authors:** Sławomir Jabłoński, Marcin Kozakiewicz

**Affiliations:** 1 Department of Thoracic Surgery, General and Oncological Surgery, Medical University of Lodz, 113 Żeromskiego St., 90-547, Łódź, Poland; 2 Department of Maxillofacial Surgery, Medical University of Lodz, 113 Żeromskiego St., 90-547, Łódź, Poland

## Abstract

**Background:**

An attempt to find a prediction method of death risk in patients affected by acute mediastinitis. There is not such a tool described in available literature for that serious disease.

**Methods:**

The study comprised 44 consecutive cases of acute mediastinitis. General anamnesis and biochemical data were included. Factor analysis was used to extract the risk characteristic for the patients. The most valuable results were obtained for 8 parameters which were selected for further statistical analysis (all collected during few hours after admission). Three factors reached Eigenvalue >1. Clinical explanations of these combined statistical factors are: Factor1 - proteinic status (serum total protein, albumin, and hemoglobin level), Factor2 - inflammatory status (white blood cells, CRP, procalcitonin), and Factor3 - general risk (age, number of coexisting diseases). Threshold values of prediction factors were estimated by means of statistical analysis (factor analysis, Statgraphics Centurion XVI).

**Results:**

The final prediction result for the patients is constructed as simultaneous evaluation of all factor scores. High probability of death should be predicted if factor 1 value decreases with simultaneous increase of factors 2 and 3. The diagnostic power of the proposed method was revealed to be high [sensitivity =90%, specificity =64%], for Factor1 [SNC = 87%, SPC = 79%]; for Factor2 [SNC = 87%, SPC = 50%] and for Factor3 [SNC = 73%, SPC = 71%].

**Conclusion:**

The proposed prediction method seems a useful emergency signal during acute mediastinitis control in affected patients.

## Introduction

Severe sepsis is still a major cause of postoperative morbidity and mortality after surgery in patients with acute mediastinitis (AM). The disease is characterized by rapid and severe course and poor prognosis despite undertaken on time aggressive surgical management and supportive treatment in the intensive care conditions. The cause of the failure of the treatment is complex. Local anatomical conditions favor the infection spread in mediastinal anatomical loose tissues and the systemic reaction to infection [1]. An association is emphasized between the increase in mortality and the delay in surgical intervention [1-4]. The etiology of AM does not remain insignificant. The best chance of survival have the patients previously healthy without earlier mediastinal pathologies in whom infection develops as a result of injury or as a complication related to endoscopic diagnostic procedures [5-7]. If the disease develops in a patient with previous history of diseases, especially of carcinoma or as the result of complications related to thoracosurgical or cardiosurgical procedures, the death risk increases [8-10]. It should be expected that a number of factors can affect the final prognosis e.g. age, etiology, delay in diagnosis, the type of surgical procedure, the kind and number of coexisting diseases, the type of a pathogen, postoperative complications and others.

The management in this severe disease could facilitate categorizing patients into appropriate risk groups in order to undertake the most optimal treatment strategy for the developing severe sepsis. Working out a simple prognostic scale on the basis of the data obtained from the medical history, clinical examination, diagnostic imaging and preliminary biochemical investigations can be one of the useful solutions. Similar prognostic scales are applied in other diseases such as e.g. acute pancreatitis: the Acute Physiology and Chronic Health Evaluation (APACHE II) scale, Ranson criteria, the Atlanta Classification of Severe Acute Pancreatitis [11-13]. Scales trying to determine the prognosis for severely sick patients have also been created e.g.: Nutritional Risk Index (NRI) [14,15] and Prognostic Inflammatory and Nutritional Index(PINI) [16].

To date no method has been available for the evaluation of the probability of recovery if a patient is affected by acute mediastinitis.

Therefore, we attempt to find a clinically useful preoperative method of predicting the prognosis for patients with AM on the basis of clinical examination and simple laboratory investigations.

## Material and methods

In the years 1998–2010, at the Department of Thoracic Surgery, General and Oncological Surgery of the Medical University of Lodz, there were treated 44 consecutive patients with AM. The study group comprised the patients fulfilling modified criteria of mediastinitis diagnosis worked out by Esterra et al. [17], which in the original version were related to descending necrotizing mediastinitis: (1) clinical manifestation of severe infection; (2) demonstration of AM etiological factors; (3) characteristic radiological picture of mediastanitis; (4) isolation of the pathogen in microbiological cultures from the mediastinal area; (5) intraoperative or postmortem documentation of mediastinitis. Exponents of sepsis in the form of: fever, tachycardia, hyperventilation and leucocytosis were observed in all patients.

The study was given an approval by the institutional Ethical Review Committee (ERC).

The age of the patients was from 19 to 83 years, mean age 52.5 years (median 54.5). There were 31 men, mean age 50,9 years (median 55) and 13 women, mean age 56.4 years (median 58). Majority of them were referred to our department after earlier treatment in other centers which had an impact on the delay in diagnosis and on appropriate surgical treatment.

The time of hospitalization was on the average about 3 weeks (23.84 ± 11.96 days, median 21.5). All patients were operated, 14 patients died. The total death rate was 31.82% (38.7% in male and 15.4% in female group).

The etiology of AM was extremely differentiated (Table 1). Iatrogenic complications were the most frequent cause of mediastinal infection. They were found in 19 patients (43.2%) and associated with esophageal and tracheal surgeries or with injuries to these organs during endoscopy or intubation. Non-iatrogenic esophageal and tracheal injuries were the cause of AM in 11 patients (25%). This group also included perforations caused by a foreign body. Descending AM was detected in 9 patients (20.4%). In 5 patients (11.4%) AM resulted from a spontaneous perforation of advanced esophageal cancer or lung cancer with infiltration to the esophagus (neoplastic etiology).

**Table 1:** Aetiology and surgical procedures in patients with acute mediastinitis

**AETIOLOGY**		**Number**	**Procedure**	**Death**
Iatrogenic (19)				
	Oesophageal endoscopy	4	PR-3E SPH-1	1
	Oesophagotomy	4	EX-2 PR-2	2
	Nissen operation	2	ESPH-1PR-1	1
	Post-intubation tracheal rupture	2	PR-2	0
	Complications of thyroid surgery	2	CRD + MDV-1 CRD + MDT-1	0
	Oesophageal rupture during intubation	1	PR-1	0
	Sternotomy complications in cardiac surgery	1	RVM-1	1
	Colonic perforation to retroperitoneal space	1	MDV-1	0
	Complications of neurosurgical procedures	1	CRD + MDV-1	0
	Complications of funnel chest surgery	1	RVM-1	0
Traumatic (11)				
	Oesophageal perforation	3	PR-3	0
	Tracheal rupture	2	PR-2	0
	Oesophageal rupture (foreign body)	3	PR-3	0
	Boerhaave syndrome	1	PR-1	1
	Burn of oesophagus with perforation	1	ESPH-1	0
	Foreign body in mediastinum (traffic accident)	1	MDS-1	0
Descending (9)				
	Dental abscess	4	DCUM- 2 CRD + MDT-2	1
	Retropharyngeal abscess	3	DCUM- 2 CRD + MDV- 1	1
	Peritonsillar abscess	2	CRD + MDT- 1 DCUM- 1	1
Neoplastic (4)				
	Esophageal spontaneous perforation after prosthesis/ stent implantation	2	EX + PN-1 EX-1	2
	Spontaneous oesophageal perforation in advanced cancer	3	PR-1 EX-1 TD-1	3

Legends of surgical procedures:

- EX: exclusion

- PR: primary repair

- ESPH: oesophagectomy

- MDT: mediastinal drainage by thoracotomy

- MDV: mediastinal drainage by videothoracoscopy

- MDS: mediastinal drainage by sternotomy

- TD: T-tube drainage

- CRD: cervical drainage

- PN: pneumonectomy

- DCUM: drainage of cervix and upper mediastinum

- RVM: revirage and mediastinal drainage

All patients underwent surgery. The time from the establishment of the diagnosis of AM to the introduction of surgical treatment ranged from 2 h to 11 days, mean 1.62 days (±1.86), median (1.0). Surgical strategy was determined individually dependently on the etiology, delay from the diagnosis establishment, local conditions and the patient’s general condition. The surgery, first of all, aimed at controlling the infection source and at limiting local inflammation by means of a wide cervical and/or mediastinal drainage through various surgical approaches. A detailed list of the performed surgical procedures and mortality rate of AM patients is presented in Table 1.

The following clinical risk features were evaluated: age, gender, etiology of coexisting diseases, delay in surgical treatment, isolated pathogens, type of surgical procedure, the number and type of postoperative complications. Then, the association between mortality rate and selected biochemical risk factors was investigated analyzing the following parameters: hemoglobin level, hematocrit, red blood cell count, leucocytosis, platelet count, serum sodium and potassium level, values of the inflammation markers: C-reactive protein (CRP) and procalcitonin (PCT) in preoperative period and on day 3 postoperatively. General and biochemical data were included (Table 2). The factors for which in statistical analysis no association was found with the prognosis were excluded from further studies.

**Table 2:** Data included into this paper from treated patients affected with acute mediastinitis

	**Age**	**Coex_diseas**	**HGB**	**WBC_pre**	**CRP_pre**	**PCT_pre**	**Proteins**	**Albumins**
Average	52,5455	1,97727	11,5677	15,2432	202,891	2,93409	57,3864	31,6773
Standard deviation	13,7595	1,42223	2,14823	5,14417	50,1198	3,92167	7,35118	3,90652

Legend: Coex_diseas – coexisting diseases; HGB – level of hemoglobin in venous blood (g/dl) WBC_pre – white blood cell count in venous blood collected before surgical treatment immediately after department admission [x10^6^/μl]; CRP_pre – C-reactive protein collected before surgical treatment immediately after department admission (mg/l); PCT_pre – procalcitonin in serum collected before surgical treatment immediately after department admission (ng/ml); Proteins – level of total serum proteins (g/l); Albumins – albumin level in serum (g/l).

### Statistical analysis

Statistical method of the factor analysis was used to extract the risk aspects for the patients (Statgraphics Centurion XVI, StatPoint Technologies, Inc. Warrenton, USA). Then, the clinical value of the extracted factors was evaluated by ANOVA, where the treatment outcome was investigated. Variances were checked by Levene’s test. As p value for this statistics was less than 0.05, Kruskal-Wallis Test was applied to check the significance.

Finally, the number of significant preoperative factors for the prognosis was reduced to 8 parameters which were grouped into 3 prognostic factors named respectively: proteinic status, inflammatory status and general status arranged dependently on their statistical power. All utilized parameters can be collected in a simple way during examination of the patient directly after admission to the ward and after laboratory investigations (within 2–3 hours).

The first factor explained as “proteinic status” informs about the initial state of protein metabolism. This parameter is composed of results of laboratory tests of blood: serum protein, albumin and hemoglobin (HGB) level.

The second factor “inflammatory status” allows to estimate the patient’s septic state on the basis of three laboratory parameters determined prior to the treatment: white blood cell count (WBC_pre), CRP value (CRP_pre), PCT value (PCT_pre).

The third factor of the prediction schema “general risk” focuses on the evaluation of the patient’s clinical state and includes only two important parameters: age (Age) and the number of coexisting diseases (Coex_disease).

Coefficients of sensitivity (SNC) and specificity (SPC) were calculated for the extracted factors to check the prediction power of the suggested method. The proposed method is designed for the prediction of recovery. Thus, the result of the test is positive (P) if the test predicts the recovery, and negative (N) if the test does not predict the recovery but i.e. “death”. Respectively, the result of the test is true (T) if the test predicts recovery when the observed result is “recovery”, and the result of the test is false (F) if the test does not predict the recovery. Therefore: TP-patient recovered and predicted as “recovery”, TN-patient died and predicted as “death”, FP-patient died but predicted as “recovery”, and FN - patient recovered but predicted as “death”. Basing on the above definitions, the suggested sensitivity and specificity coefficients equations are:

Sensitivity coefficient: $\text{SNC}=\frac{\text{TP}}{\text{T}P+FN}\times 100\%$

Specificity coefficient: $\text{SNC}=\frac{\text{TP}}{TP+FN}\times 100\%$

## Results

Three factors have been extracted as statistically requested (Eigenvalue > 1), they are presented in Table 3. Together they account for over 69% of the variability in the original data. The initial communality estimates have been set to assume that all of the variability in the data is due to common factors (principal components method). Next, factor loading matrix was calculated. In order to simplify the clinical explanation of the factors, the rotation of the matrix was performed. Table 4 shows the parameters for equations, which estimate the common factors after rotation has been performed. Basing on those scores in the next statistical step, the factor (rotated) equations were constructed:

(1) $$\begin{array}{c} \text{Factor}1=0,712131\text{HGB}+0,854481\text{Proteins}-0,131796\text{Coex}\_\text{diseas}+0,00534419\\ \text{WBC}\_\text{pre}-0,141942\text{Age}+0,908303\text{Albumins}-0,651832\text{CRP}\_\text{pre}-\\ 0,560482\text{PCT}\_\text{pre} \end{array}$$

(2) $$\begin{array}{c} \text{Factor}2=0,152337\text{HGB}-0,0461529\text{Proteins}-0,0604516\text{Coex}\_\text{diseas}+\\ 0,914729\text{WBC}\_\text{pre}+0,263779\text{Age}-0,0949298\text{Albumins}+0,514794\text{CRP}\_\text{pre}+\\ 0,371643\text{PCT}\_\text{pre} \end{array}$$

(3) $$\begin{array}{c} \text{F}3=-0,243032\text{HGB}-0,0418942\text{Proteins}+0,863627\text{Coex}\_\text{diseas}+\\ 0,108861\text{WBC}\_\text{pre}+0,685527\text{Age}-0,167625\text{Albumins}+0,0364827\text{CRP}\_\text{pre}+\\ 0,141625\text{PCT}\_\text{pre} \end{array}$$

where the values of the variables (x) in the equations are standardized by subtracting their means (μ) and dividing by their standard deviations (σ). It also shows the estimated communalities, which can be interpreted as estimating the proportion of the variability in each variable attributable to the extracted factors.

**Table 3:** Factor Analysis – presentation of the factors

**Factor** ** Number**	**Eigenvalue**	**Percent of** ** Variance**	**Cumulative** ** Percentage**	**Initial** **Communality**
1	3,31109	41,389	41,389	1,0
2	1,16325	14,541	55,929	1,0
3	1,04991	13,124	69,053	1,0
4	0,754858	9,436	78,489	1,0
5	0,682004	8,525	87,014	1,0
6	0,540662	6,758	93,772	1,0
7	0,358296	4,479	98,251	1,0
8	0,139929	1,749	100,000	1,0

Note: for 3 factors the Eigenvalue is >1.

**Table 4:** Factor loading matrix after varimax rotation

**Parameter**	**Factor score coefficients**			**Estimated** **Communality**	**Specific** **Variance**
	Factor1	Factor2	Factor3		
HGB	0,712131	0,152337	−0,243032	0,589401	0,410599
Proteins	0,854481	−0,0461529	−0,0418942	0,734023	0,265977
Coex_diseas	−0,131796	−0,0604516	0,863627	0,766875	0,233125
WBC_pre	0,00534419	0,914729	0,108861	0,848609	0,151391
Age	−0,141942	0,263779	0,685527	0,559674	0,440326
Albumins	0,908303	−0,0949298	−0,167625	0,862124	0,137876
CRP_pre	−0,651832	0,514794	0,0364827	0,691229	0,308771
PCT_pre	−0,560482	0,371643	0,141625	0,472317	0,527683

**Figure 1:**
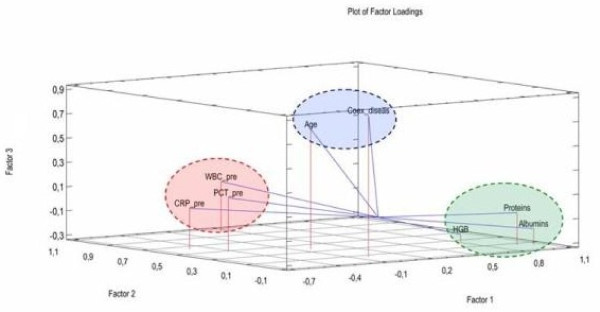
Plot of final factor loading after matrix rotation.

Visual presentation of extracted factors is shown in Figure 1. Final factor scores calculated for all factors included into this study, together with easy explanation of their meanings are presented in Table 5.

**Table 5:** Factor scores

**Case**	**Observed outcome**	**Factor1**	**Factor2**	**Factor3**	**Classification result**
		Proteinic status	Inflammatory status	General risk	
		Recovery Prediction for > −1.4*	Recovery Prediction for <1.0*	Recovery Prediction for <0.4*	
1	Death	−8,61293	1,97822	2,03692	TN
2	Recovery	**−1,89787**	**1,01016**	**0,60735**	FN
3	Death	−5,67083	1,17312	3,18046	TN
4	Recovery	2,96689	0,0611059	−1,30167	TP
5	Death	−4,61678	**0,759947**	1,84367	TN
6	Death	−3,58174	3,35379	2,15131	TN
7	Recovery	1,24868	−0,0408765	0,219901	TP
8	Recovery	**−2,55507**	0,0101163	−0,12325	FN
9	Recovery	0,858724	0,588775	0,151806	TP
10	Recovery	**−1,95238**	0,0573994	0,216068	FN
11	Recovery	3,64292	−0,704072	−0,684944	TP
12	Recovery	2,6163	−1,34394	−2,25436	TP
13	Death	−4,19034	4,84986	0,915751	TN
14	Recovery	1,82354	0,0636333	−1,26561	TP
15	Recovery	3,03511	**1,12721**	−0,649727	TP
16	Recovery	3,37817	−1,38079	−2,2192	TP
17	Recovery	0,580244	−0,316079	−1,61708	TP
18	Death	−1,56375	**−2,27389**	**−0,389642**	TN
19	Death	−1,78795	**−0,0187813**	**−0,789484**	TN
20	Recovery	0,57392	**1,00331**	−0,714067	TP
21	Recovery	2,86891	−0,0531427	−1,05936	TP
22	Recovery	0,343636	0,293051	**0,970888**	TP
23	Recovery	1,14208	−0,965971	−1,6126	TP
24	Recovery	5,51418	−1,06023	−3,28449	TP
25	Death	−3,25473	1,29683	1,1493	TN
26	Death	−2,12645	2,29104	0,529981	TN
27	Recovery	2,29387	0,0084471	**1,6481**	TP
28	Death	**0,907509**	**−0,6706**	3,7267	FP
29	Recovery	1,29283	−1,69442	0,299441	TP
30	Recovery	3,45795	−1,35408	−2,1628	TP
31	Death	**2,96653**	**−1,47445**	**−0,86775**	FP
32	Recovery	0,0323576	−1,55881	**0,509574**	TP
33	Recovery	1,0745	0,26778	0,334441	TP
34	Death	−2,52481	2,36734	1,19426	TN
35	Recovery	1,32141	**1,05543**	**0,824733**	TP
36	Recovery	1,29592	−1,62119	−2,8627	TP
37	Death	−5,05654	**0,832591**	1,11692	TN
38	Recovery	2,11607	−0,960466	−0,634111	TP
39	Recovery	**−1,4526**	0,511999	1,41191	FN
40	Recovery	−0,871523	−1,93215	−0,779669	TP
41	Death	**3,87668**	**−2,0661**	**−0,280317**	FP
42	Recovery	−1,30349	−0,759654	**0,880723**	TP
43	Recovery	1,76721	−1,49925	−1,44593	TP
44	Recovery	0,0236466	−1,21219	**1,07856**	TP

**Figure 2:**
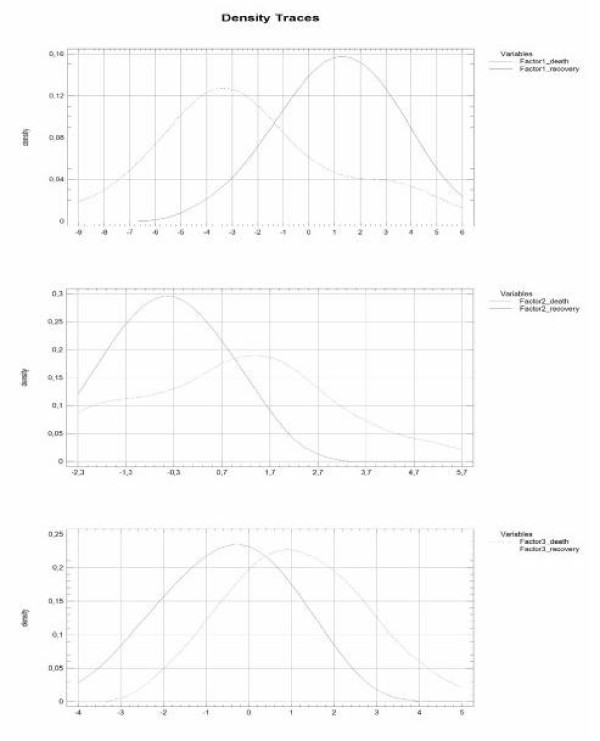
**Comparison of Proteinic status (Factor 1), Inflammatory status (Factor 2), and General risk (factor 3) in subpopulation of recovery and lethal outcome of acute mediastinitis.** The difference is statistically significant.

This table shows the factor scores (prognostic) for each patient from our data file (observation). * − level of dichotomic division of subpopulation: recovery and death, see cross of the lines in density traces (Figure 2). **Bold**-lack of agreement between prediction and observation. Classification result: TP-true positive, TN-true negative, FP-false positive, FN-false negative.

There are three separate groups: HGB + Proteins + Albumim, WBC_pre + CRP_pre + PCT_pre, and Age + Coex_diseas.

According to the above, clinical meaning of the factors was established. Factor1 (F1) is “proteinic status” of the patient, Factor2 (F2) – “inflammatory status”, and Factor3 (F3) – “general risk” understood as the composite of age and number of coexisting diseases. The statistics of these factors in the presented clinical series is shown in Table 6.

**Table 6:** Final statistics of factor scores

**Parameter**	**Factor1**	**Factor2**	**Factor3**
	Proteinic status	Inflammatory status	General risk
Count	44	44	44
Average	9,55 × 10^-8^	5,11 × 10^-7^	1,36 × 10^-7^
Median	0,72	−0,005	0,18
Standard deviation	2,98	1,50	1,52
Minimum	−8,61	−2,27	−3,28
Maximum	5,51	4,85	3,73
Range	14,13	7,12	7,01
Standardized skewness	−1,88	2,64	0,26
Standardized kurtosis	0,45	1,94	−0,01

Finally, clinical verification was done by checking the extracted factors value in our series of patients concerning the outcome of the treatment as recovery or death (Table 7). ANOVA results are presented in Figure 2 for F1 (F = 21.78, p < 0.0001; Levene`s test = 4.0978, p < 0.05; Kruskal-Wallis test = 11.9168, p < 0.001), for F2 (F = 8.45, p < 0.01; Levene`s test = 8.9967, p < 0.01; Kruskal-Wallis test = 4.9168, p < 0.05), and for F3 (F = 14.18, p < 0.001; Levene`s test = 0.0001, p = 0.9929).

**Table 7:** Final statistics of subgroups (factor scores)

	**Factor1**		**Factor2**		**Factor3**	
	Proteinic status		Inflammatory status		General risk	
	death	recovery	death	recovery	death	recovery
Count	14	30	14	30	14	30
Average	−2,52	1,17	0,89	−0,41	1,11	−0,52
Median	−2,89	1,27	1,00	−0,18	1,13	−0,64
Standard deviation	3,36	1,89	2,06	0,93	1,41	1,30
Minimum	−8,61	−2,56	−2,27	−1,93	−0,87	−3,28
Maximum	3,88	5,51	4,85	1,13	3,73	1,65
Range	12,49	8,07	7,12	3,06	4,59	4,93
Stnd. skewness	0,64	−0,19	0,16	0,18	0,41	−0,70
Stnd. kurtosis	0,20	−0,18	−0,28	−1,38	−0,43	−0,79

The final number of extracted factors was three. Furthermore, the coefficients of sensitivity and specificity were calculated for each factor (for F1: SNC = 87%, SPC = 79%; for F2: SNC = 87%, SPC = 50%; for F3: SNC = 73%, SPC = 71%), and next the prevalence test classification (TP, TN, FP, FN) was performed to establish the whole prognostic power of the method: SNC = 90%, SPC = 64%.

**Figure 3:**
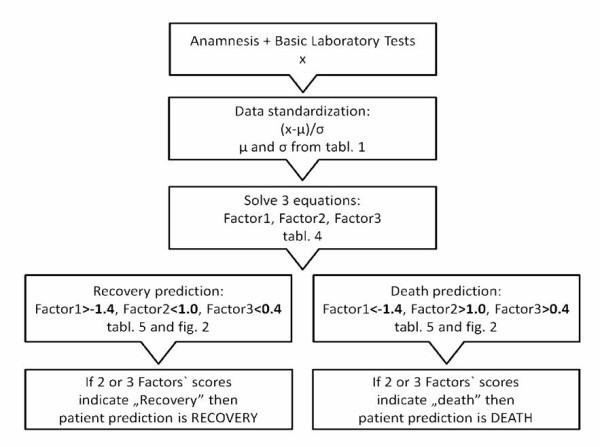
Schema of the application of the recovery prediction method.

The schema of the proposed prediction method application is presented in Figure 3.

The probability of recovery increases when F1 is higher. In other words, when “proteinic status” is worse the risk of death is higher. As far as the “inflammatory status” (F2) is concerned, in our series, lower scores are observed in recovery outcome cases. The same trend is noticed in the analysis of “general risk” (i.e. F3). When plot (Figure 2) of “proteinic status” is analyzed, the value dividing recovery outcome from death is approximately −1,4 (F1). It should be understood as high probability of the patient’s recovery if the score is higher than −1.4. In case of “inflammatory status” the caesura is located around +1.0 (F2). The prediction of survival is for patients with the score lower than +1.0. Respectively, “general risk” (F3) score lower than +0.4 is a prediction of recovery outcome (as presented in tab.5). The predictable result based on F1 is most of all in compliance with the observed result of the treatment (only 7 variances/44 results). The variances result from the application of 3 factors. It should be known that if 8 parameters are subject of analysis, the whole explanation of variability is possible with 8 factors. The same is visible in density traces (Figure 2) where full strict dichotomic separation of recovery from death outcome subpopulations is impossible. That kind of mutually penetrating subpopulations is often observed in biological sciences.

## Discussion

Early recognition of septic complications, information about sepsis severity and thus, the ability to predict the prognosis can have a significant impact on the treatment strategy in AM. Access to such data can be of importance in establishing the urgency and type of surgical intervention, monitoring in postoperative period, necessity for repair, the kind of antibiotic-therapy and supportive treatment. In medicine, numerous prognostic scales have been created allowing to assess the prognosis in selected pathological states. However, in available literature we have not found a scale related to acute mediastinitis. Most probably it results from rare prevalence of this disease and difficulty in gathering appropriately rich material within one medical centre. The proposed prognostic method, based on the evaluation of 8 simple and easy to obtain parameters compiled in the form of 3 factors, allows dichotomic categorization of patients into 2 groups as regards the predicted prognosis: survival or death. When the calculated values of individual factors are combined, it is easy to distinguish within first few hours of hospitalization the patients whose prognosis is worse than that of the others. Obviously, the selection of proper parameters for the estimation of the predicted prognosis in the course of AM can be the subject of discussion.

In practice the first information about the patient’s general condition is obtained during taking the history data. At this stage we can obtain the data regarding patient’s age and coexisting diseases which in the proposed prognostic scale are important for calculating factor 3 values. In critically ill patients with sepsis, older age and coexisting diseases are associated with poor prognosis [18-20]. There are several prognostic scales considering the effectof coexisting diseases on the prognosis. The best known are: Charlson Comorbidity Index (CCI), Davies (Stokes) score and Index of Coexisting Diseases (ICED). They are widely applied in the patients dialyzed due to renal failure [21-24]. Charlson scale, which estimates similar parameters as our scale but it is based on different methodology, is used most frequently. It takes into account 19 coexisting diseases which are assigned with a score. CCI includes age as one of the evaluated elements and the age scores are counted according to the following scheme: 1 score for each decade over 40 years of age. The total score enables to predict the prognosis [25]. It was demonstrated in C-Y Wang’s study that higher value of CCI (>2) in patients treated surgically due to stage I of lung cancer was associated with higher mortality rate than in the group of patients with lower number of comorbidities; CCI < 2 [26]. The proposed by us prognostic scale is different because the data on the general state (F3) are only one of three estimated elements. If after substituting the data concerning age and coexisting diseases for the given formula for “F3” we obtain the value < +0.4, there increases the chance for the patient’s survival. F3 is important for the whole scale but according to our calculations it has a lower diagnostic value compared to the remaining two factors (SNC = 73%, SPC = 71%). Anticipating the questions why there were not taken into account such obvious clinical risk factors as: etiology, delay in diagnosis or the type of pathogen, we want to explain that the introduction of these data, to our surprise, led in consequence to the weakening of the algorithm diagnostic value. It seems that additional parameters can act the opposite way in the whole pool. Presumably, the factor analysis eliminated less reliable variables leaving those which presented the highest predictive power in the proposed algorithm. For instance, the delay in diagnosis and the time of the introduction of the surgical treatment are not unequivocal parameters. It is worth emphasizing that majority of the patients were hospitalized earlier on other wards, where initially no proper diagnosis was established. Furthermore, they were then subjected to surgical procedures the effect of which could sometimes deteriorate their condition and sometimes improve it partially. Similar remarks concern the bacterial flora which changed in the course of the treatment and finally its distribution was the effect of coincidence, antibiotic therapy and/or infection. It was impossible to classify such internally unstable parameters by the method of factor analysis and attempts of their inclusion into the algorithm had a negative effect on the accuracy of the prediction.

Laboratory investigations are important elements of the proposed algorithm. The determination of other risk factors, found in already mentioned 2 factors: “proteinic status” and “inflammatory status” using 6 simple biochemical tests, supplements our prognostic method. F1 determines the initial state of the patient’s protein metabolism on the basisof 3 parameters: total protein, albumin and HGB level. Malnutrition and hypoproteinemia are distinctly associated with increased death rate due to infection and neoplastic disease [27,28]. An objective estimation of malnutrition and protein metabolism is usually difficult, it is based on clinical observation, determination of BMI and biochemical investigations [29]. Among biochemical markers albumin level is most frequently used in malnutrition assessment. Hypoalbuminemia is associated with malnutrition and the decrease of protein level because liver reduces albumin production in favor of more important plasma proteins [16]. In 1988 Busby et al., first described the Nutritional Risk Index (NRI) to score the severity of postoperative complications [14,15]. It combines two nutritional indicators (albumin and weight loss), which are strictly correlated with higher morbidity and mortality risk in the population of elderly patients [30]. The need of determining ideal body weight, which is difficult in elderly or critically ill patients, is one of the limitations of this scale. Thus, it became necessary to find a formula enabling to calculate ideal body weight, which led to creation of a new, more objective tool – the Geriatric Nutritional Risk Index (GNRI) [31]. Basing on the performed analysis we have demonstrated that there is also a need for inclusion of the hemoglobin level into the prognostic scale. It was included into the markers estimating “proteinic status”. The presence of anemia is a known factor affecting poor prognosis, particularly in combination with other diseases [32,33]. The studies of Welch et al. demonstrated that general death risk increases with the decrease of HGB concentration and even benign forms of anemia can be associated with the increase of the death risk [34]. The advantage of the suggested prognostic method is the determination of protein metabolism in simpler way than in NRI or GNRI basing only on biochemical tests which is of importance in patients in critical condition. The obtained high diagnostic value for “proteinic status”, corresponding with the final prognosis (SNC = 87%, SPC = 79%) should be . If the value of F1 calculated on the basis of the formula is lower than −1.4, it means a high death risk for the patient.

We are convinced that in the case of infectious diseases limitation to the assessment of protein metabolism, age and co-existing diseases is not sufficient for the prediction of the prognosis. It seems natural to extend the prognostic scale including biochemical markers of inflammation. White blood cell count (WBC) is the oldest widely used marker. It should be reminded that WBC value is one of the criteria of SIRS and sepsis diagnosis [35]. Fever in combination with elevated WBC count is a quick and cheap way of infection diagnosis but its low diagnostic value is its basic limitation [36]. This parameter in combination with other inflammatory markers still has a wide clinical application both in the diagnosis and monitoring of the results of the treatment. CRP remains one of the most important classic markers for inflammation. It is included into sensitive but little specific acute phase proteins, the level of which increases in inflammation and malignancy [37,38]. It has been confirmed that initial CRP values were directly associated with total mortality rate in neoplastic disease [39]. However, Matson et al. paid attention to the fact that “normal” plasma CRP level in critically ill patients is rarely the same as in healthy population [40]. The post-mortem studies demonstrated that in patients with cachexia related to malignant carcinoma, in the case of extensive tumor necrosis, significant deviations were observed in the behavior of acute phase proteins [41]. That is why in these cases the determination of CRP alone can appear to be insufficient in the monitoring of inflammation. PCT is a biochemical marker extremely useful in the diagnosis and differentiation of severe infections and septic complications [42-44]. The increase of PCT concentration induced by bacterial toxins (with preserved insensitivity to other pro-inflammatory stimuli) and close relation between serum PCT concentration and infection severity are the most important properties of this marker [45,46]. Taking into account the above mentioned properties we have included serum PCT concentration into F2 evaluation. We think that simultaneous determination of three biochemical markers will enable more precise and more objective assessment of “inflammatory status” eliminating errors resulting from heterogeneity of the investigated group and known deviations in the behavior of the selected markers in some critical morbid states.

Among the prognostic scales using inflammatory state markers we have not found any similar to ours. Our scale is unique due to the combination of biochemical data of inflammation with simultaneous assessment of the patient’s general condition and protein metabolism. Ingenbleek and Carpentier Prognostic Inflammatory and Nutritional Index (PINI) deserves attention [16]. The scale is based on the evaluation of 4 parameters: 2 markers of malnutrition: albumin and prealbumin, and 2 markers of inflammatory state: CRP and α1acid glycoprotein (AAG). This scoring system may predict morbidity or mortality in hospitalized patients [24]. The normal PINI level in healthy population is <1. The value of PINI (>1) is associated with poor prognosis [16,47]. PINI has been found to be a reliable indicator of both nutritional status and prognosis in trauma, burns and infection [48,49] and lately in cancer [50]. PINI is slightly similar to the scale proposed by us, as it considers 2 of 3 analyzed groups of risk factors. In our investigations we did not determine AAG, which is not a marker commonly used in clinical practice in our country, and prealbumin due to its susceptibility to nutrition inhibition, which always occurs in the course of the treatment of AM patients. Other authors also confirmed that nutritional state can affect inflammatory response in patients with advanced carcinoma and the results of PINI prognostic scale [51,52]. Wunder et al. presented an interesting attempt of working out an independent indicator of early prediction of death in sepsis [53]. The authors, analyzing 33 patients with sepsis of different etiology, noticed that the deviations of the values of PCT and Acute Physiology and Chronic Health Evaluation (APACHE II) were correlated with poor prognosis. Novotny et al. carried out similar studies on a larger group of 160 patients with sepsis resulting from peritonitis or mediastinitis after an anastomotic leak and perforation of a hollow organ [54]. It should be noted that the clinical material presented in this study was to a great extent similar to our material. The authors, owing to combination of both indicators and calculations with the use of binary logistic regression analysis, were able to identify the groups of high and low death risk. In a multivariate analysis, both PCT and APACHE III score were identified as independent, early predictive indicators of sepsis lethality. While 71% of the high-risk patients died of sepsis, 77% of patients assigned to the low-risk group survived the septic complication (sensitivity 71%, specificity 77%) [54]. To compare, the diagnostic value for “inflammatory status” in the suggested method obtained higher sensitivity (87%) but lower specificity (50%). Poor prognosis should be suspected in patients whose value of F2 calculated from the formula is <1.0.

High death rate in the course of AM points to the need of further studies. Rare prevalence of the disease and high differentiation of the material within one medical centre are the limitations. Thus, introduction of multicentre register of the patients should be taken into consideration. A detailed analysis of the investigated cases in a large representative group of patients can have an influence on the determination of risk factors and on the improvement of the prognosis in patients treated surgically due to AM.

## Conclusion

We do hope that the proposed prognostic method has a chance to be introduced into the clinical practice which can contribute to the modification of the treatment of patients with AM. It is based on mathematical assessment of own material and devoid of subjective interpretation. Its most important advantages are: inclusion into the assessment of 2 simple clinical data and 6 biochemical tests which can be obtained within first 2–3 hours after the patient’s admission to hospital (duration of laboratory investigations), low costs and simple interpretation of the results. We think that the construction of the method, based on the evaluation of 3 groups of risk factors determining inflammatory, proteinic and general status, will be less sensitive to difficult to foresee deviations of the values of biochemical markers associated with the impact of factors such as: malnutrition, bacteriological etiology, comorbidities, surgical complications and others. To simplify the calculations, the scale can be prepared in a form of automatic electronic “calculator” which provides a ready result after entering appropriate data. The result proving poor prognosis should induce to more aggressive surgical treatment and to modification of antibiotic-therapy and supportive treatment.

## Consent

Written informed consent was obtained from the patient for publication of this report and any accompanying images.

## Abbreviations

AM: acute mediastinitis; APACHE: Acute Physiology and Chronic Health Evaluation; NRI: Nutritional Risk Index; PINI: Prognostic Inflammatory and Nutritional Index; CRP: C-reactive protein; PCT: Procalcitonin; HGB: Hemoglobin; WBC: White blood cell; pre: Preoperative; Coex_disease: coexisting diseases; SNC: Sensitivity; SPC: Specificity; P: Positive; N: Negative; T: True; F: False; TP: True positive; TN: True negative; FP: False positive; FN: False negative; F1: Factor1; F2: Factor2; F3: Factor3; CCI: Charlson Comorbidity Index; ICED: Index of Coexisting Diseases; BMI: Body mass index; GNRI: Geriatric Nutritional Risk Index; SIRS: Systemic Inflammatory Response Syndrome; PINI: Prognostic Inflammatory and Nutritional Index; AAG: α1acid glycoprotein.

## Competing interests

The authors declare that they have no competing interests.

## Authors’ contribution

JS and KM are equally engaged into the study: Study design, data collection, statistical analysis, data interpretation, manuscript preparation, literature search, and funds collection. Both authors read and approved the final manuscript.

## References

[B1] Marty-Ané CH Berthet JP Alric P Management of descending necrotizing mediastinitis: an aggressive treatment for an aggressive disease Ann Thorac Surg 1999 68 212 217 10421143 10.1016/s0003-4975(99)00453-1

[B2] Muir AD White J McGuigan JA McManus KG Grahamoraz AN Treatment and outcomes of oesophageal perforation in a tertiary referral centre Eur J Cardiothorac Surg 2003 23 799 804 12754036 10.1016/s1010-7940(03)00050-2

[B3] Reeder LB DeFilippi VJ Ferguson MK Current results of therapy for esophageal perforation Am J Surg 1995 169 615 617 7771627 10.1016/s0002-9610(99)80232-3

[B4] Freeman RK Vallières E Verrier ED Karmy-Jones R Wood DE Descending necrotizing mediastinitis: an analysis of the effects of serial surgical debridement on patient mortality J Thorac Cardiovasc Surg 2000 119 260 267 10649201 10.1016/S0022-5223(00)70181-4

[B5] Bufkin BL Miller JI Mansour KA Esophageal perforation: emphasis on management Ann Thorac Surg 1996 61 1447 1452 8633957 10.1016/0003-4975(96)00053-7

[B6] Dubost C Kaswin D Duranteau A Jehanno C Kaswin R Esophageal perforation during attempted endotracheal intubation J Thorac Cardiovasc Surg 1979 78 44 51 449386

[B7] Akman C Kantarci F Cetinkaya S Imaging in mediastinitis: a systematic review based on aetiology Clin Radiol 2004 59 573 585 15208062 10.1016/j.crad.2003.12.001

[B8] El Oakley RM Wright JE Postoperative mediastinitis: classification and management Ann Thorac Surg 1996 61 1030 1036 8619682 10.1016/0003-4975(95)01035-1

[B9] Schroeyers P Wellens F Degrieck I De Geest R Van Praet F Vermeulen Y Vanermen H Aggressive primary treatment for poststernotomy acute mediastinitis: our experience with omental- and muscle flaps surgery Eur J Cardiothorac Surg 2001 20 743 746 11574218 10.1016/s1010-7940(01)00873-9

[B10] Jones WG Ginsberg RJ Esophageal perforation: a continuing challenge Ann Thorac Surg 1992 53 534 543 1489367 10.1016/0003-4975(92)90294-e

[B11] Leung TK Lee CM Lin SY Chen HC Wang HJ Shen LK Balthazar computed tomography severity index is superior to Ranson criteria and APACHE II scoring system in predicting acute pancreatitis outcome World J Gastroenterol 2005 11 6049 6052 16273623 10.3748/wjg.v11.i38.6049 PMC4436733

[B12] Blamey SL Imrie CW O’Neill J Gilmour WH Carter DC Prognostic factors in acute pancreatitis Gut 1984 25 1340 1346 6510766 10.1136/gut.25.12.1340 PMC1420197

[B13] Bradley EL A clinically based classification system for acute pancreatitis. Summary of the International Symposium on Acute Pancreatitis, Atlanta, Ga., September 11 through 13, 1992 Arch Surg 1993 128 586 590 8489394 10.1001/archsurg.1993.01420170122019

[B14] Buzby GP Knox LS Crosby LO Study protocol: a randomized clinical trialof total parenteral nutrition in malnourished surgical patients Am J Clin Nutr 1988 47 366 381 3124598 10.1093/ajcn/47.2.366

[B15] Buzby GP Williford WO Peterson OL A randomized clinical trial of total parenteral nutrition in malnourished surgical patients: the rationale and impact of previous clinical trials and pilot study on protocol design Am J Clin Nutr 1988 47 357 365 3124597 10.1093/ajcn/47.2.357

[B16] Ingenbleek Y Carpentier YA A prognostic inflammatory and nutritional index scoring critically ill patients Int J Vitam Nutr Res 1985 55 91 101 3922909

[B17] Estrera AS Lanay MJ Grisham JM Descending necrotizing mediastinitis Surg Gynecol Obstet 1983 157 545 552 6648776

[B18] Martin GS Mannino DM Moss M The effect of age on the development and outcome of adult sepsis Crit Care Med 2006 34 15 21 16374151 10.1097/01.ccm.0000194535.82812.ba

[B19] Yang Y Yang KS Hsann YM Lim V Ong BC The effect of comorbidity and age on hospital mortality and length of stay in patients with sepsis J Crit Care 2010 25 398 405 19836195 10.1016/j.jcrc.2009.09.001

[B20] Azoulay E Adrie C De Lassence A Determinants of postintensive care unit mortality: a prospective multicenter study Crit Care Med 2003 31 428 432 12576947 10.1097/01.CCM.0000048622.01013.88

[B21] Fried L Bernardini J Piraino B Charlson Comorbidity Index as a predictor of outcomes in incident peritoneal dialysis patients Am J Kidney Dis 2001 37 337 342 11157375 10.1053/ajkd.2001.21300

[B22] Beddhu S Bruns FJ Saul M Seddon P Zeidel ML A simple comorbidity scale predicts clinical outcomes and costs in dialysis patients Am J Med 2000 108 609 613 10856407 10.1016/s0002-9343(00)00371-5

[B23] Athienites NV Miskulin DC Fernandez G Bunnapradist S Simon G Landa M Comorbidity assessment in hemodialysis and peritoneal dialysis using the Index of Coexistent Disease Semin Dial 2000 13 320 326 11014695 10.1046/j.1525-139x.2000.00095.x

[B24] Davies SJ Russell L Bryan J Phillips L Russell GI Comorbidity, urea kinetics and appetite in continuous ambulatory peritoneal dialysis patients: their interrelationship and prediction of survival Am J Kidney Dis 1995 26 353 361 7645541 10.1016/0272-6386(95)90657-6

[B25] Charlson ME Pompei P Ales KL MacKenzie CR A new method of classifying prognostic comorbidity in longitudinal studies: development and validation J Chronic Dis 1987 5 373 383 3558716 10.1016/0021-9681(87)90171-8

[B26] Wang C-Y Lin Y-S Tzao C Lee H-C Huang M-H Hsu W-H Hsu H-S Comparison of Charlson comorbidity index and Kaplan—Feinstein index in patients with stage I lung cancer after surgical resection Eur J Cardiothorac Surg 2007 32 877 881 17920921 10.1016/j.ejcts.2007.09.008

[B27] Marti J Armadans L Vaque J Segura F Schwartz S Protein-calorie malnutrition and lymphocytopenia as predictors of hospital infection in the elderly Med Clin (Barc) 2001 116 446 450 11333701 10.1016/s0025-7753(01)71865-9

[B28] Chen MK Souba WW Copeland EM Nutritional support of the surgical oncology patient Hematol Oncol Clin North Am 1991 5 125 145 1902827

[B29] Reynolds TM Stokes A Russell L Assessment of a prognostic biochemical indicator of nutrition and inflammation for identification of pressure ulcer risk J Clin Pathol 2006 59 3 308 310 16505284 10.1136/jcp.2005.029405 PMC1860338

[B30] Naber HJ de Bree A Schermer TRJ Specificity of indexes of malnutrition when applied to apparently healthy people: the effect of age Am J Clin Nutr 1997 65 1721 1725 9174466 10.1093/ajcn/65.6.1721

[B31] Bouillanne O Morineau G Dupont C Coulombel I Vincent J-P Nicolis I Benazeth S Cynober L Aussel Ch Geriatric Nutritional Risk Index: a new index for evaluating at-risk elderly medical patients Am J Clin Nutr 2005 82 777 783 16210706 10.1093/ajcn/82.4.777

[B32] Carson JL Noveck H Berlin JA Gould SA Mortality and morbidity in patients with very low postoperative Hb levels who decline blood transfusion Transfusion 2002 42 7 812 818 12375651 10.1046/j.1537-2995.2002.00123.x

[B33] Garson JL Duff A Poses RM Berlin JA Spence RK Trout R Noveck H Strom BL Effect of anaemia and cardiovascular disease on surgical mortality and morbidity Lancet 1996 348 1055 1060 8874456 10.1016/S0140-6736(96)04330-9

[B34] Welch HG Mehan KR Goodnough LT Prudent strategies for elective red blood cell transfusion Ann Intern Med 1992 116 393 402 1736773 10.7326/0003-4819-116-5-393

[B35] American College of Chest Physicians/Society of Critical Care Medicine Consensus Conference definitions for sepsis and organ failure and guidelines on the use of innovative therapies in sepsis-Members of the American College of Chest Physicians/Society of Critical Care Medicine Consensus Conference Committee Crit Care Med 1992 20 864 874 1597042

[B36] Weitzman M Diagnostic utility of white blood cell and differential cell counts Am J Dis Child 1975 129 1183 1189 1190139 10.1001/archpedi.1975.02120470033008

[B37] Okamura JM Miyagi JM Terada K Hokama Y Potential clinical applications of C-reactive protein J Clin Lab Anal 1990 4 231 235 2112596 10.1002/jcla.1860040316

[B38] Clark BA Mayhew JL An inexpensive method of determining body composition by underwater weighing Br J Nutr 1979 42 173 183 7448489 10.1136/bjsm.14.4.225 PMC1858813

[B39] Balkwill F Mantovani A Inflammation and cancer: back to Virchow? Lancet 2001 357 539 545 11229684 10.1016/S0140-6736(00)04046-0

[B40] Matson A Soni N Sheldon J C-reactive protein as a diagnostic test of sepsis in the critically ill Anaesth Intensive Care 1991 19 182 186 2069236 10.1177/0310057X9101900204

[B41] Warren S The immediate causes of death in cancer Am J Med Sciences 1932 184 610 615

[B42] Brunkhorst FM Wegscheider K Forycki ZF Brunkhorst R Procalcitonin for early diagnosis and differentiation of SIRS, sepsis, severe sepsis, and septic shock Intensive Care Med 2000 26 Suppl 2 148 252 18470710 10.1007/BF02900728

[B43] Bertsch T Richter A Hofheinz H Bohm C Hartel M Aufenanger J Procalcitonin.A new marker for acute phase reaction in acute pancreatitis Langenbecks Arch Chir 1997 382 367 372 9498210 10.1007/s004230050081

[B44] de Werra I Jaccard C Corradin SB Cytokines, nitrite/nitrate, soluble tumour necrosis factor receptors, and procalcitonin concentrations: comparisons in patients with septic shock, cardiogenic shock, and bacterial pneumonia Crit Care Med 1997 25 607 613 9142024 10.1097/00003246-199704000-00009

[B45] Dalton H Procalticonin: a predictor of lung injury attributable to sepsis Crit Care Med 1999 25 2304 2308 10548234 10.1097/00003246-199910000-00049

[B46] Whang KT Vath SD Becker KL Procalticonin and proinflamatory cytokine interactions in sepsis Shock 2000 4 73 78 10909897 10.1097/00024382-200014010-00013

[B47] Perier C Granouillet R Chamson A Gonthier R Frey J Nutritional markers, acute phase reactants and tissue inhibitor of matrix metalloproteinase 1 in elderly patients with pressure sores Gerontology 2002 48 298 301 12169795 10.1159/000065253

[B48] Gottschlich MM Baumer T Jenkins M Khoury J Warden GD The prognostic value of nutritional and inflammatory indices in patients with burns J Burn Care Rehabil 1992 13 105 113 1572837

[B49] Bonnefoy M Ayzac L Ingenbleek Y Kostka T Boisson RC Bienvenu J Usefulness of the prognostic inflammatory and nutritional index (PINI) in hospitalized elderly patients Int J Vitam Nutr Res 1998 68 189 195 9637950

[B50] Walsh D Mahmoud F Barna B Assessment of nutritional status and prognosis in advanced cancer: interleukin-6, C-reactive protein, and the prognostic and inflammatory nutritional index Support Care Cancer 2003 11 60 62 12527956 10.1007/s00520-002-0390-z

[B51] Slaviero KA Clarke SJ Rivory LP Inflammatory response: an nrecognized sourceof variability in the pharmacokinetics and pharmacodynamics of cancer chemotherapy Lancet Oncol 2003 4 224 232 12681266 10.1016/s1470-2045(03)01034-9

[B52] Rivory LP Slaviero K Clarke SJ Hepatic cytochrome P450 3A drug metabolism isreduced in cancer patients with an acute-phase response Br J Cancer 2002 87 277 280 12177794 10.1038/sj.bjc.6600448 PMC2364233

[B53] Wunder C Eichelbronner O Roewer N Are IL-6, IL-10 and PCT plasma concentrations reliable for outcome prediction in severe sepsis? A comparison with APACHE III and SAPS II Inflamm Res 2004 53 158 163 15060722 10.1007/s00011-003-1239-3

[B54] Novotny A Emanuel K Matevossian E Kriner M Ulm K Bartels H Holzmann B Weighardt H Siwert J-R Use of procalcitonin for early prediction of lethal outcome of postoperative sepsis Am J Surg 2007 194 35 39 17560906 10.1016/j.amjsurg.2006.10.026

